# Going ‘Above and Beyond’: Are Those High in Autistic Traits Less Pro-social?

**DOI:** 10.1007/s10803-014-2056-3

**Published:** 2014-02-13

**Authors:** Leila Jameel, Karishma Vyas, Giulia Bellesi, Victoria Roberts, Shelley Channon

**Affiliations:** Department of Cognitive, Perceptual and Brain Sciences, University College London (UCL), Bedford Way Building, Gower Street, London, WC1E 6BT UK

**Keywords:** Autistic traits, Pro-social behaviour, Empathy, Perspective-taking, Theory of mind

## Abstract

Few studies have explored how the cognitive differences associated with autistic spectrum disorder translate into everyday social behaviour. This study investigated pro-social behaviour in students scoring high and low on the autism-spectrum quotient (AQ), using a novel scenario task: ‘Above and Beyond’. Each scenario involved an opportunity to behave pro-socially, and thus required balancing the needs of a character against participants’ own interests. High AQ participants both generated responses and selected courses of action that were less pro-social than those of the low AQ group. For actions of low pro-social value they gave higher self-satisfaction ratings; conversely, they gave lower self-satisfaction ratings for high pro-social actions. The implications for everyday functioning are considered for those with high autistic traits.

## Introduction

Despite an abundance of work examining cognitive performance in those with autistic spectrum disorders (ASDs), there is a paucity of literature exploring how the cognitive profiles identified translate into everyday social functioning. ‘Pro-social behaviour’ refers to intentional acts designed to help others, and is thought to be important for both society and the individual (Eisenberg and Miller 1987; Eisenberg et al. 1998). Examples of pro-social actions include helping, sharing, donating, co-operating and volunteering (Brief and Motowidlo 1986). Behaving pro-socially has been found to aid social bonding, to have a positive impact on social adjustment, self-esteem, and to contribute towards psychological wellbeing and physical health (Coie et al. 1990; Eisenberg et al. 1998; Puffer 1987). Within a group, pro-social action is thought to maximise benefits for the ‘greater good’ (Hoffman 2001). Although ASD is associated with impaired social performance, there is relatively little work examining pro-social behaviour in this population. Some evidence from studies of charitable giving suggests that those with ASD donated less and showed reduced preference for charities benefiting other people, as compared to controls (Lin et al. 2012), and were less influenced by the presence of an observer (Izuma et al. 2011).

Various authors have emphasised the role of empathy in motivating socially sensitive behaviour (Eisenberg 2007; Minio-Paluello et al. 2009), and it has been positively associated with engagement in pro-social behaviour (Eisenberg and Miller 1987; Sze et al. 2011). For example, feeling more empathy has been linked to a greater concern for others’ welfare and more helping behaviours (Batson 1991). Although the distinction has not always been clearly delineated, empathy as a motivating force for pro-social behaviour is postulated to involve both emotional and perspective-taking mechanisms (Eisenberg and Miller 1987). Perspective-taking is considered to be the cognitive component of empathy, also termed ‘theory of mind’ or ‘mentalising’ (Blair 2008; Rogers et al. 2007), and all refer to the ability to attribute and infer the content of others’ mental states by taking their perspective (Premack and Woodruff 1978). By contrast, emotional empathy refers to the mirroring of others’ emotional states (Eisenberg and Miller 1987; Hoffman 2001). Vicariously invoked feelings of distress or discomfort and increased physiological arousal when witnessing someone in need may play a motivating role in pro-social behaviour (Batson 1987; Coke et al. 1978; Eisenberg 2003; Eisenberg et al. 1989; Eisenberg and Miller 1987). Thus, acting on behalf of others in need is a ‘self’-orientated action, which reduces the vicarious empathic arousal experienced (Schaller and Cialdini 1988). On the other hand, acting on mental state apprehension of others’ needs on the basis of perspective-taking is an ‘other’-orientated process.

Both perspective-taking and emotional empathy are thought to contribute to successful social functioning (Blair 2008; Rogers et al. 2007), but it has been posited that emotional empathy is intact whilst perspective-taking is impaired in ASD (Blair 2008); individuals with ASD are thus thought to be able to feel for others, but not to understand them. A deficit in perspective-taking is well supported by a range of evidence and accounts more fully than other cognitive theories for the social impairments characteristic of ASD (Happe et al. 2006b; Spek et al. 2010). By 4 years of age, most typically developing children begin to understand and explain false belief scenarios (Happe 1995) involving predicting others’ behaviour on the basis of their false belief. Children with ASD are slower to develop these abilities (Happe 1995), and struggle with more abstract and less explicit perspective-taking tasks (Heavey et al. 2000). In adults with ASD, perspective-taking difficulties are often tested at a more subtle level in empirical tasks, such as failure to understand and infer the motives, intentions and emotions of characters in stories (Happe 1994; Spek et al. 2010). Failure to detect faux-pas in social situations has also been identified, demonstrating an inability to appreciate and predict the responses of others (Spek et al. 2010; Stone et al. 1998).

Perspective-taking has also been linked to measures of social and interpersonal skills (Dawson and Fernald 1987). Individuals who fail false belief tasks (Baron-Cohen et al. 1985) have been found to show less insightful social behaviour and poorer verbal communication skills (Frith 1994). Furthermore, performance on measures of perspective-taking have been associated with skills required for appropriate social behaviour, including ability to maintain conversation and respond appropriately (Hale and Tager-Flusberg 2005), to contribute novel information (Capps et al. 1998), to understand non-literal language (Martin and MacDonald 2004), and to identify others’ embarrassment (Hiller and Allinson 2002). Individuals with ASD have been found to be impaired in all of these areas.

Although the concept of impaired perspective-taking is well supported, the notion of intact emotional empathy in ASD remains disputed, with some studies finding evidence of impairment (Minio-Paluello et al. 2009; Singer et al. 2004). A broken ‘mirror neuron system’ (MNS) has been hypothesised to be the cause of the poor social skills and perspective-taking difficulties characteristic of those with ASD (Iacoboni and Dapretto 2006; Oberman and Ramachandran 2007; Williams et al. 2001). The MNS is defined as the regions in the inferior parietal and inferior frontal cortex that respond both when an individual performs an action, and when observing another’s action (Rizzolatti and Craighero 2004). The MNS is thought to facilitate matching the actions of the self to those of others’, thereby supporting the ability to infer others’ intentions (Hamilton and Grafton 2006). In turn, the ability to understand others’ actions and goals might underlie social abilities including perspective-taking (Gallese and Goldman 1998; Gallese et al. 2004). Whilst the MNS has received a lot of attention, the extent to which it is able to mediate more complex social abilities such as perspective-taking remains poorly understood (Southgate and Hamilton 2008). In any case, mirroring another’s actions, and thus possibly their intentions, may not be sufficient to invoke emotional empathy, since it is also necessary to project any feelings thereby induced onto the observed other, and then to understand them in the context of the other’s mental state. Consistent with this, some work both in healthy adults (Samson et al. 2010), and in a patient with a lesion to the prefrontal and temporal brain regions (Samson et al. 2005) has suggested that difficulty with perspective-taking tasks may result from a failure to inhibit one’s own perspective in favour of another’s. Conflicting evidence for intact emotional empathy may therefore be explained by some capacity of those with ASD to resonate emotionally with others, but to do so from a ‘self’ stance (Frith and de Vignemont 2005; Minio-Paluello et al. 2009).

It may well be that individuals with ASD are only capable of resonating emotionally with others if it is made explicit what they are thinking or feeling. If perspective-taking skills are necessary to mediate the adequate identification of others’ needs, then it would be expected that individuals with ASD would be less successful in behaving pro-socially. Whilst a core deficit in perspective-taking in individuals with ASD is well supported, there is a need for a fine-grained approach to understanding the ramifications for everyday behaviour, which may in turn facilitate more precise insights into the nature of their difficulties and inform interventions such as social skills training (Channon et al. 2012).

The revisions in DSM-5, in which differential diagnoses of autism, Asperger Syndrome, and so on, are subsumed into one single diagnostic category of ASD (APA 2013) are consistent with a continuum view of autism. There is now evidence that autistic traits are present to varying degrees in the general population (Baron-Cohen et al. 2001). One commonly used method for assessing autistic traits is Baron-Cohen et al.’s (2001) self-report questionnaire, the ‘Autism-Spectrum Quotient’ (AQ). The AQ was designed for use with individuals of typical intelligence and assesses core traits linked to ASD. It was developed using a clinical sample of individuals diagnosed with high-functioning ASD, a control sample of healthy volunteers and a student sample. All those scoring highly reported significant social impairments, such as difficulty forming and maintaining relationships with peers, social isolation and being the victims of bullying.

Recent studies have adopted the methodology of comparing groups with different levels of autistic traits, and this has proved fruitful in elucidating cognitive and behavioural differences in the broader phenotype, including visuospatial skills (Almeida et al. 2012; Bayliss and Kritikos 2011; Grinter et al. 2009), biological motion processing (van Boxtel and Lu 2013), the identification of animate versus inanimate objects (Burnett and Jellema 2013), emotion processing (Cooper et al. 2013; Poljac et al. 2012), and personality correlates (Austin 2005). However, very little work has examined autistic traits in relation to social behaviour. In one such study, Hudson et al. (2012) investigated learning of social information by requiring participants to observe two characters whose non-verbal cues, such as facial expression and gaze, conveyed either a positive or negative disposition. In a subsequent gaze-cueing task, only those with low AQ scores were found to have learned this information as reflected by their speeded responses, whereas those with high AQ scores showed no evidence of learning the characters’ dispositions; the authors attributed this to impairment in implicit learning. In another study, Yang and Baillargeon (2013) found that participants with more autistic traits performed less well in a novel social task involving rating the appropriateness of a character’s responses.

The present study adopted a trait-based approach to compare pro-social behaviour in those high and low in autistic traits on the AQ, using a novel scenario-based task; ‘Above and Beyond’. In this task participants were asked to read a series of scenarios, each involving a character in need. In order to assess ability both to generate and judge appropriate pro-social responses, they first generated a free response to each of the scenarios, and then selected a response from a choice of three alternatives, representing low, medium and high pro-social courses of action. This allowed for examination of whether reducing the task demands also reduced any group differences in pro-social behaviour. In two previous studies examining real-life-type problem solving also using social scenario-based tasks, those with ASD were found to display difficulty in generating problem solutions, but not in judging alternatives (Channon et al. 2001, 2014). On this basis, it was hypothesised that the high AQ group may have been able to identify which was the best option when presented with alternatives, but not to produce it spontaneously. It was predicted that those with high AQ scores would generate courses of action that were less pro-social than those with low AQ scores, and that they might also choose less pro-social courses of action when selecting amongst alternatives. Finally, participants were asked to give satisfaction ratings for each possible course of action from both their own perspective (self) and that of the main character (other). The self- versus other-satisfaction ratings were expected to reveal potential difficulties in taking the characters’ perspectives, whereby the high AQ group would give lower estimates than the low AQ group of the characters’ satisfaction when they performed actions of high pro-social value, and conversely, would give higher estimates of the characters’ satisfaction when they performed actions of low pro-social value. In addition, it was predicted that the high AQ group would experience less personal satisfaction for going ‘above and beyond’ (performing actions of high pro-social value) than the low AQ group.

## Methods

### Screening Phase

#### The Autism-Spectrum Quotient

The AQ (Baron-Cohen et al. 2001) is a brief, self-administered questionnaire that measures personality traits associated with the autistic spectrum in adults of typical intelligence. It consists of 50 statements rated on a four point Likert scale (1 = definitely agree; 4 = definitely disagree) covering different aspects of autistic symptomatology (APA 2000; Rutter 1978; Wing and Gould 1979); social skill, attention switching, attention to detail, communication and imagination. Total AQ trait scores thus range from a minimum of 0 to a maximum of 50. Approximately half the items are worded to produce a ‘disagree’ response, and half an ‘agree’ response. It has been found to have good internal consistency and construct validity, strong test–retest reliability, and robust self versus parent report reliability (Baron-Cohen et al. 2001).

#### Participants and Procedure

The study was granted ethical approval from the UCL Research Ethics Committee. An opportunistic sample of 573 full-time university students (43 % male) who were fluent in English and aged 18 or over (mean age 20 years) was recruited for the screening phase of the study. All participants provided informed consent before completing the AQ (Baron-Cohen et al. 2001). As an incentive, participants were entered into a prize draw and informed that they might be invited to take part in the second phase of the study, for which they would be paid. Total AQ scores were calculated for the whole sample. Since AQ traits are more common in males than in females (Baron-Cohen et al. 2001), participants within the highest-scoring and lowest-scoring 10 % of males and the highest-scoring and lowest-scoring 10 % of females were contacted via email and invited to take part in the second stage. These formed the high AQ and low AQ participant groups for the experimental phase of the study.

### Experimental Phase

#### Participants and Procedure

Of those contacted from the screening phase, 27 (14 female, 13 male) individuals from the upper range and 24 (12 female, 12 male) individuals from the lower range agreed to take part in the experimental phase of the study, forming two groups of high AQ and low AQ participants. AQ scores ranged from 25 to 43 in the high AQ group (25–43 for male participants, and 26–37 for female participants), and 3–10 in the low AQ group (4–10 for male participants, and 3–9 for female participants). A *t* test confirmed that AQ scores differed significantly between groups, *t*(1,49) = 24.42, *p* = .0001; mean AQ scores were 30.70 (SD = 4.33), and 6.83 (SD = 2.16) for the high and low AQ groups respectively. The groups did not differ significantly in age, *t*(1,49) = .495, *p* = .623; mean age was 20.37 (2.71) and 20.79 (3.36) for the high and low groups respectively. All participants reported the degree subject that they were studying (see Table 1), and choice of subject differed significantly between groups. There was a higher predominance of scientific degree subjects in both the high AQ participants who were contacted to take part in the experimental phase of the study, and in those who formed the high AQ group in the experimental phase, relative to their low AQ counterparts: contacted: *t*(1,155) = 3.92; *p* = .0001, tested: *t*(1,49) = 2.22; *p* = .031.

**Table 1:** Degree subject breakdown for participants contacted and tested

Participants contacted	Low AQ group (N = 42)	High AQ group (N = 57)	Significance (*p* = .05)
Science %	26 %	60 %	.0001
Non-science %	74 %	40 %	–

Participants tested	Low AQ group (N = 24)	High AQ group (N = 27)	Significance (*p* = .05)
Science %	29 %	59.25 %	.031
Non-science %	71 %	40.75 %	–

‘Science’ was defined as the natural and mathematical sciences, and also included allied disciplines such as biomedical science, chemical engineering, genetics and pharmacy

‘Non-science’ included all other social sciences and humanities

All participants were tested individually, and provided written informed consent before completing the ‘Above and Beyond’ task. They were also asked to complete a brief health-screening questionnaire that asked about any serious accidents or illnesses, psychological or emotional difficulties; in practice no exclusions were required. Participants were paid for their efforts.

#### The ‘Above and Beyond’ Task^1^

The ‘Above and Beyond’ task was designed to assess propensity to behave pro-socially in everyday situations, and the lengths to which individuals are willing to go to help others. A range of scenarios was devised and piloted with healthy volunteers of different ages, social backgrounds and ethnicity in order to refine the items and develop the scoring system. The final set consisted of 10 brief scenarios describing social situations, involving a main character in need of help, where only the participant was potentially available to help them. Each scenario required a difficult social judgment with respect to balancing the needs of the character against their own interests. The character was male in half the scenarios, and female in the other half, and the type of relationship and social context varied across scenarios to reflect a natural range of situations. To control for order effects, two different scenario orders were created and counterbalanced within each group.

The scenarios were presented on paper, and participants were taken through an example before completing the 10 experimental items. Scenarios and questions were presented in separate booklets such that relevant scenarios remained on display throughout task performance in order to minimise any memory demands. Each scenario was followed by four questions. Participants were first asked to generate responses for what they would do in the situation, and were then asked which course of action they would be most likely to follow when presented with a choice of three. These were designed to represent low, medium and high pro-social actions, requiring increasing effort on the part of the participants. Participants were also required to rate satisfaction with each action from their own and the main character’s perspective.

#### Example Scenario

“You are walking down an empty side street when a man trips over in front of you and falls down heavily on the pavement. You are in a rush to get to work on time for a meeting.”

#### Questions for Each Scenario

##### *Generation of pro-social response:*

What would you do in this situation?

##### *Selection of pro-social action:*

Which of the following would you most likely to do?

(*Low*): Carry on walking.

(*Medium*): Help him up and carry on walking.

(*High*): Help him up and offer to take him to sit down on a nearby bench.

##### *Satisfaction ratings:*

1. *Self-perspective (participant):* On a scale of 1–10, where 1 represents ‘not at all pleased’ and 10 represents ‘very pleased’, how ‘pleased’ would you feel if you chose to do the following?
*[rate low, medium and high actions]*
2. *Other-perspective (character):* On a scale of 1–10, where 1 represents ‘not at all pleased’ and 10 represents ‘very pleased’, how ‘pleased’ would he feel if you chose to do the following?
*[rate low, medium and high actions]*

### Scoring

#### Generation of Pro-social Responses

Scoring of verbal responses for each scenario was in accordance with their pro-social value; one point for low, two points for medium, and three points for high pro-social value. Low pro-social actions were those involving little effort on the participant’s part, tending to prioritise their own needs over others. Medium pro-social actions involved making significant effort to help another, but within limits as to the personal cost. High pro-social actions went ‘above and beyond’ in helping others to their own disadvantage. In the example shown above, where a man has fallen over, a response classified as low pro-social effort involved making little or no attempt to stop and help the man (e.g. “Continue rushing to work on time, assume someone else will help him.”). A response classified as medium pro-social effort described stopping to help the man up and some attempt to offer further assistance, but made it clear that the participant was not prepared to be late for their meeting (e.g. “Check if he is okay and if I can call for him first of all. Try to keep in mind that I am in a rush.”). A response classified as high pro-social effort indicated that the participant was prepared to be late for their meeting if required (e.g. “Stop and help the man up, see if he needs medical attention. My meeting can’t be that important—probably phone to say I might be a bit late.”).

The responses were classified by a rater who was not blind to group membership, and by a second, blind independent rater. There was an inter-rater agreement rate of 94.23 %; all disagreements were resolved by a third party adjudicator (also blind to group membership). Participant scores were then summed across all 10 scenarios (range 10–30).

#### Selection of Pro-social Actions

Participants were awarded a score of 1 for choosing the lowest pro-social actions, 2 for choosing medium pro-social actions and 3 for choosing the highest pro-social actions. Participant scores were then summed across all 10 scenarios (range 10–30).

#### Self- Versus Other-Satisfaction Ratings

For each scenario, participants rated satisfaction from both their own (self) and the main characters’ (other) perspectives on a scale of 1–10, where higher scores indicated greater satisfaction. Scores were summed across all 10 scenarios (range 10–100), creating 6 scores; low, medium and high satisfaction for self-perspective; and low, medium and high satisfaction for other-perspective. An overall self-satisfaction difference score was then calculated (high pro-social actions score minus low pro-social actions score); an overall other-satisfaction difference score was calculated on the same basis.

## Results

### Data Analysis

Means and standard deviations (SD) for each of the measures below are presented in Table 2. A significance level of .05 was adopted, with a stricter level (.05/3 = .017) for post hoc tests to control for multiple comparisons.

**Table 2:** Mean percentage scores and standard deviations for all measures for the ‘Above and Beyond’ task

	Low AQ group (N = 24) M (SD)	High AQ group (N = 27) M (SD)	Significance (*p* = .05)	Effect Size
Generation of pro-social response (%)				
Total quality	81.94 (6.59)	67.40 (11.82)	.0001	1.52
Low pro-social	10.42 (9.08)	28.15 (20.20)	.0001	1.13
Medium pro-social	30.83 (14.42)	40.37 (17.86)	.043	0.53
High pro-social	56.36 (13.64)	41.00 (18.53)	.013	0.95
Selection of pro-social action (%)				
Total score	83.89 (7.39)	72.22 (10.97)	.0001	1.25
Low action	6.25 (6.47)	18.51 (16.10)	.0001	1.82
Medium action	36.25 (15.82)	46.29 (17.35)	.037	0.60
High action	57.50 (17.99)	35.19 (20.82)	.0001	1.15
Self-(participant) perspective ratings (%)				
High-low satisfaction difference	27.46 (14.54)	7.74 (20.33)	.0001	1.12
Low action	45.46 (10.44)	54.22 (11.26)	.006	0.81
Medium action	70.67 (9.52)	66.52 (11.19)	–	–
High action	72.92 (7.30)	61.96 (16.64)	.005	0.85
Other-(character) perspective ratings (%)				
High-low satisfaction difference	44.91 (8.61)	39.89 (10.50)	.070	0.52
Low action	40.33 (7.38)	45.04 (9.40)	–	–
Medium action	70.21 (7.30)	70.14 (6.72)	–	–
High action	85.25 (6.24)	84.93 (7.18)	–	–

### The ‘Above and Beyond’ Task

#### Generation of Pro-social Responses

A t-test was used to compare the high and low AQ groups on the total score for generation of pro-social responses. The high AQ group scored significantly lower than the low AQ group, *t*(1,49) = 5.332, *p* = .0001, suggesting that their responses were less often classified as pro-social.

Post-hoc *t* tests were conducted to examine the pattern underlying this overall difference in score. The groups did not differ in their generation of medium pro-social responses, *t*(1,49) = 2.081, *p* = .043, but did significantly differ in their generation of low and high pro-social responses, whereby the high AQ group generated fewer high pro-social responses, *t*(1,49) = 2.64, *p* = .013, and more low pro-social responses, *t*(1,49) = 3.97, *p* = .0001.

#### Selection of Pro-social Actions

The high and low AQ groups were compared on total scores for selection of pro-social actions. The high AQ group was found to behave significantly less pro-socially overall than the low AQ group, *t*(1,49) = 4.392, *p* = .0001, suggesting that they chose fewer high pro-social actions and more low pro-social actions.

Further *t* tests were carried out to examine choices of low, medium and high pro-social actions separately, summed across scenarios. Using a strict significance level of .017, the groups did not differ on the medium pro-social actions, *t*(1,49) = 3.49, *p* = .037; the high AQ group was found to choose significantly more low pro-social actions, *t* (1,49) = 3.49, *p* = .0001, and significantly fewer high pro-social actions, *t*(1,49) = 4.07, *p* = .0001.

#### Self- Versus Other-Satisfaction Ratings

A repeated measures ANOVA was conducted, using the overall high-low satisfaction difference scores to compare groups for self-perspectives (participant) versus other-perspectives (character). There was one between-group factor (high vs. low AQ), and one within-group factor (self-satisfaction difference score vs. other-satisfaction difference score). There were significant main effects of perspective, *F*(1,49) = 84.82, *p* = .0001, and group, *F*(1,49) = 17.08, *p* = .0001, and a significant perspective by group interaction *F*(1,49) = 7.43, *p* = .009.

Post-hoc *t* tests were conducted to compare the two groups for overall self- and other-satisfaction difference scores separately, using a strict significance level (*p* = .017). The groups did not differ significantly for other-satisfaction difference scores, *t*(1,49) = 1.86, *p* = .070, but did show a significant difference for self-satisfaction difference scores, *t*(1,49) = 3.94, *p* = .0001. Comparison of mean scores revealed that for self-satisfaction scores the high AQ group differentiated very little between low and high courses of action; they also tended to rate satisfaction for high pro-social actions lower than the low AQ group, *t*(1,48) = 2.98, *p* = .005, and rated satisfaction for low pro-social actions higher than the low AQ group, *t*(1,48) = 2.87, *p* = .006.

## Discussion

The present study examined how autistic traits translate into everyday pro-social behaviour. It employed a novel scenario-based task describing everyday situations, in which a main character required help, to assess the generation and selection of pro-social responses in groups with high and low self-reported autistic traits. The pattern of results supported the prediction that the high AQ group would behave less pro-socially overall, since high AQ participants generated verbal responses that were significantly less pro-social in quality than those of their low AQ counterparts. It was also hypothesised that any group differences might be ameliorated when the need to generate responses was removed, and participants were simply required to select responses from a choice of three possible courses of action, but this was not supported. Thus, the high AQ participants were less pro-social both in their spontaneous generation of responses and in their selection of actions from alternatives. In addition, participants rated satisfaction from their own perspective and from those of the main characters. The high AQ group did not differ from the low AQ group in ratings of the characters’ satisfaction, but they did differ in self-satisfaction ratings, where they differentiated less between the degrees of pro-social behaviour. They tended to express greater satisfaction for performing low pro-social actions and lesser satisfaction for performing high pro-social actions.

Both asking people to generate their own responses and to choose amongst alternatives differentiated the groups significantly. With respect to the generation of pro-social content, the high AQ group’s verbal responses contained fewer classified as high pro-social, and more classified as low pro-social (see methods for a scoring example) relative to the low AQ group, with similar numbers of responses that were of medium pro-social value. For instance, in one of the scenarios participants were asked to decide what they would do if a friend, upset that her partner had just broken up with her, rang at an inconvenient time. Medium pro-social responses (e.g. “Calm her down, help her and make her feel better over the phone.”) were effective in responding to the main characters’ needs, but did not incur significant personal costs. The high AQ group was less successful at generating responses of high pro-social value that went ‘above and beyond’, failing to prioritise others’ needs over consideration of their own (*e.g. Talk to her for as long as she wanted and offer to go round. I would try and make sure she is okay*—*me having a quiet night in isn’t as important.*). The high AQ group also made more verbal responses that were low in pro-social value and gave little help to the main character (*e.g. Try to end the phone call as soon as possible, or wait for the answer machine to get it.*).

From a theoretical viewpoint, a number of different accounts might be pertinent to the present findings. There are three traditional explanations that are believed, to some extent, to account for the cognitive deficits characteristic of ASD. Firstly, performance deficits in ASD have been attributed to impaired perspective-taking (cognitive empathy) with preserved emotional empathy, as previously discussed. Before examining this, the remaining two, weak central coherence and executive dysfunction will be considered. These are more commonly used to explain the non-social symptoms of ASD, but may also mediate social impairments. Other potential contributory factors considered below include acquired aspects of performance such as the role of social knowledge and social learning.

### Contribution of Social Knowledge

One important consideration is that everyday-type tasks such as ‘Above and Beyond’ may involve drawing on previously acquired social knowledge. Here, the term social knowledge is used to refer to the unwritten conventions and rules that govern societal functioning. For instance, taking the scenario where someone falls over as you are walking by, the unwritten ‘rule’ could be stated as “You should stop and help someone who might be injured”. For the scenario where a friend has broken up with her partner, the unwritten rule might be said to be “You should comfort a friend who is upset”. It has been suggested that knowledge stores relating to prior social experience may be more limited in those with ASD (Channon et al. 2001). This may result from a lack of exposure to relevant social situations and/or a reduced capacity to acquire relevant social knowledge. Lack of exposure to social situations may come about because individuals with ASD actively avoid social encounters (Richer 1976), which is often attributed to a sense of anxiety associated with such experiences (White et al. 2011). Whilst this study did not measure social engagement specifically, the high AQ participants were found to be more likely to have chosen a scientific degree subject, and less likely to study more socially-oriented subjects such as social sciences or the humanities. This finding replicates that of with previous work (Baron-Cohen et al. 2001), and is also consistent with the finding that those with a systemising-driven versus empathising-driven cognitive style are more likely to study for science degrees (Carroll and Chiew 2006; Manson and Winterbottom 2012). Moreover, the AQ (Baron-Cohen et al. 2001) includes various statements (e.g.“I would rather go to a library than to a party.” and “I prefer to do things with others rather than on my own.”) that are likely to elicit agreement and disagreement respectively in high trait individuals, indicating a pattern consistent with that reported in the ASD population. Thus, it is possible that the high AQ group engaged less in social interaction, and had fewer opportunities to gain relevant social knowledge.

Reduced capacity to acquire such knowledge may also play a part, since those with ASD are well known to be impaired in skills including pretend play (Travis and Sigman 1998), which offers children opportunities to engage in complex social negotiations and to practice social roles. With respect to the ‘Above and Beyond’ task, the low AQ group appeared to show greater compliance with social expectations by acting more pro-socially overall, often inconveniencing themselves in the process. It is possible that reduced social knowledge in the high AQ participants meant that were less aware of these expectations or may have felt less pressure to comply, resulting in behaviour that was less pro-social. Furthermore, even if their social knowledge was intact, they may have been less motivated to apply it. It is well established that individuals with ASD show diminished responses to social rewards, and this has been related to reduced social learning (Zeeland et al. 2010). In the present study, insensitivity to reward may account in part for the high AQ group’s reduced pro-social behaviour. Potential sources of reward include possible reciprocal future actions by the characters in need, and intrinsic reward through satisfaction gained by helping the characters. Thus, there are several ways in which inadequate social knowledge and/or application of such knowledge could have influenced performance.

### Contribution of Non-social Models

Weak central coherence (Frith 1989, 2003; Happe 1999; Happe and Frith 2006) postulates that those with ASD tend to process information in a piecemeal as opposed to holistic manner, involving an enhanced focus on local features at the expense of contextual details (Joliffe and Baron-Cohen 1999; Shah and Frith 1993). This may have implications for social functioning via a failure to appreciate the social context of a situation (De Martino et al. 2008; Lawson et al. 2004). With respect to performance on the ‘Above and Beyond’ task, increased attention to the details of the scenarios may have resulted in the high AQ group experiencing difficulty integrating the information to form an understanding of the wider social context. For instance, those in the high AQ group may have concentrated only on the details relevant to their own perspective when generating responses and making their choices, leading to less pro-social behaviour overall.

The term ‘executive function’ encompasses a wide range of skills involved in the higher order control of behaviour for the pursuit of a specific goal or aim (White et al. 2009), and there is evidence that those with ASD are impaired on a range of executive tasks (for a review see Hill 2004). This theory again may also impact on social functioning and communication (Happe et al. 2006a) as a result of impaired ability to evaluate relevant aspects of social situations, generate and plan appropriate responses and appreciate the social consequences of these. Thus, in relation to the current pattern of findings, one possible explanation of the group differences is difficulty in generating pro-social courses of action. This could account for the finding that those with high AQ traits behaved less pro-socially when asked what they would do in the situations. However, if this explanation were exhaustive, then the groups should not have differed in their pro-social behaviour once the demands of generating responses were removed, and they were instead provided with cues (alternative courses of action) and required to select from these. In fact, a tendency for those in the high AQ group to behave less pro-socially was not confined to the generation of responses, but was also evident in their judgments of alternative responses. The high AQ participants chose fewer high pro-social and more low pro-social courses of action. Thus, an explanation of performance differences between the groups in terms of the inability of the high AQ participants to formulate or bring to mind pro-social courses of action is not adequate to account for the findings.

Executive deficits could also manifest as reduced inhibition or capacity to control impulse reactions in people with high AQ scores, resulting in less pro-social responses, or as failure to use appropriate strategies to search knowledge stores for relevant experience and to evaluate accurately possible future outcomes of different courses of action (Channon et al. 2001). The high AQ group may have acted impulsively in their own interests, at the expense of considering the possible benefits of pro-social behaviour for the both the main character in the short-term and themselves in the long-term. This explanation is consistent with some evidence indicating that inability to consider other perspectives may result from an executive failure to inhibit one’s own perspective (Samson et al. 2005, 2010). Imagination is also known to be impaired in those with ASD, and may contribute to a general deficit in generating and executing plans (Harris and Leevers 2000). Thus, it is possible that the high AQ participants were less able to imagine themselves in the scenarios, and to generate appropriate solutions or to envisage the consequences of following alternative courses of action.

### Contribution of Perspective-Taking

Whilst non-social models including WCC and executive dysfunction could be applicable to the present pattern of findings, impaired perspective-taking (Baron-Cohen et al. 1985) is perhaps the most plausible cognitive model since it focuses directly on the social deficits associated with ASD. Pro-social behaviour is thought to involve the identification of others’ perspectives and needs (Batson 1991). Failure to appreciate the characters’ needs and feelings in the current scenarios might have operated to reduce motivation to act pro-socially, leading in turn to fewer pro-social verbal responses and choices of actions.

Perhaps the most direct evidence with respect to perspective-taking in the present study comes from the self- and other-satisfaction ratings. As hypothesised, the groups differed significantly in their self-ratings, where the range was narrower for the high versus the low AQ group. The high AQ group rated their satisfaction higher for performing low pro-social actions of little benefit to the main character (e.g. for the falling over scenario: *carry on walking*; for the break up with partner scenario: *hang up as quickly as possible*), and rated their satisfaction lower for high pro-social actions, which went ‘above and beyond’ the social expectation to help the character (e.g. for the falling over scenario: *stop to help the man up and offer him additional aid*; e.g. for the break up with partner scenario: *offer to go and visit your friend*). In contrast with predictions, the high AQ group was not found to differ from the low AQ group when rating satisfaction from the perspective of the main characters. Both groups judged low pro-social actions to be the least satisfactory, and high pro-social actions to be the most satisfactory for the characters.

Why did the groups differ for self- but not other-satisfaction ratings? Impaired perspective-taking is one possible explanation of this; other possibilities include reduced emotional empathy, and/or reduced capacity to experience or recognise their own emotions. With respect to perspective-taking, the lack of a group difference on the other-satisfaction ratings may indicate intact ability in the high AQ group, but this seems unlikely in the context of the well-documented difficulties in the literature (e.g. Happe 1994; Spek et al. 2010). Alternatively, the current task may not have been sufficiently sensitive to reveal any perspective-taking difficulties. Scenarios were designed to examine how a reduction in task demands (generation vs. selection of pro-social responses) may relate to pro-social behaviour. Thus, for the selection component of the task, the layout systematically presented the low pro-social actions first and the high pro-social actions last, to make the pro-social values salient. It is therefore conceivable that the perspective-taking difficulties of the high AQ group were ‘masked’ by this when they were asked to rate the main characters’ satisfaction. For successful social functioning, individuals are likely to draw upon their social knowledge when considering others’ perspectives and empathising with their needs, thereby facilitating a flexible response to novel situations. Non-intuitive social knowledge may fail to support effective social interaction, as it is over-reliant on rigid rules and tends to be slowly and clumsily applied (Bowler 1992). Abnormal patterns of social behaviour may therefore be observed in those with impaired perspective-taking, even when relevant social knowledge is available to them. Thus, the high AQ group may have used task cues and other deliberately learned social rules to rate the main characters’ satisfaction. Future studies could improve the sensitivity of the ‘Above and Beyond’ task by adding an extra component requiring the participant to provide more specific information about their perception of the characters’ perspectives. For instance, participants could be asked to describe what the characters might think and feel in response to their actions.

Although perspective-taking impairment in the high AQ group may not have been detected by the other-satisfaction ratings, it may nevertheless have mediated group differences on other components of the task. Difficulties in understanding how the characters would view their own actions may have influenced their action choices and ratings of their own satisfaction, reflecting prioritisation of their own interests over those of the characters, even when they could readily gauge that a different action might be more beneficial to the main character. Perspective-taking difficulties could thus account for reduced pro-social behaviour and diminished personal gratification for going “above and beyond”. A reliance on salient task cues may have obviated the need for emotional identification with the characters, highlighting the difficulty of dissociating cognitive from emotional aspects of empathy. Without an intuitive appraisal of the characters’ needs, the high AQ participants may not have identified with them emotionally and may thus have experienced less satisfaction for helping them.

Turning to the other possible explanations for the present pattern of findings, impaired emotional empathy for others in the context of intact perspective-taking in the high AQ group could hypothetically account for the pattern of impaired self- and intact other-satisfaction ratings. However, in the light of the substantial body of literature pointing towards the opposite pattern, tending to find impaired perspective-taking with intact emotional empathy in those with ASD (Blair 2008; Singer et al. 2004), this seems unlikely. More plausible as an explanation of the findings is the notion of reduced capacity to experience or recognise their own emotions in the high AQ group. At the simplest level, reduced capacity to experience emotions could lead to a narrower range of self-satisfaction ratings across the three levels of pro-social action. There is also evidence that individuals with ASD have difficulty identifying and describing their own emotions (Hill et al. 2004). Whilst higher-functioning individuals on the spectrum show capacity to recognise and express basic emotions (i.e. happiness, sadness, and anger) difficulty with more complex or self-conscious emotions (i.e. pride and embarrassment) has been reported (Capps et al. 1992), and has been linked to the well-documented impairments with taking others’ perspectives (Frith and Happe 1999). Furthermore, a recent study found that as compared to the low AQ group, high AQ scorers experienced selective difficulty in recognising emotions, and required expressions of higher intensity to do so correctly (Poljac et al. 2012). Thus, personal experience of satisfaction for behaving pro-socially on the ‘Above and Beyond’ task may represent a self-conscious emotional experience involving an appreciation of the social context, including appraisal of the characters’ needs and responses to help.

### Implications for Everyday Social Functioning

This study established that there are performance differences on the ‘Above and Beyond’ task in what might be considered a sub-clinical population of those high versus low in autistic traits. This methodology could be extended further by including a broader range of autistic traits to examine whether there is a consistent relationship along the continuum between number of traits and social performance. Further work is also required in order to assess how the pattern of findings from the high trait group relates to a clinical sample of those diagnosed with ASD.

Although the present findings cannot be used to make definitive claims about the theoretical processes underlying the performance patterns for the high and low AQ groups, this study does highlight the potential utility of tasks of this nature in relation to social behaviour. Pro-social behaviour is linked to a number of benefits, both to the individual and to society as a whole (Coie et al. 1990; Eisenberg et al. 1998; Hoffman 2001; Puffer 1987). Regardless of the specific nature of the mechanisms that may underlie performance differences, using everyday life-type tasks to study social behaviour in those with autistic traits helps us to appreciate the everyday difficulties associated with ASD. It is of course possible that, as a result of social desirability effects, the low AQ group in fact made more pro-social choices in this task than they would actually display in real life. By contrast, the high AQ group might have been more honest in describing their real-life actions, since individuals with ASD have been found to display diminished sensitivity to protecting their social reputations (Izuma et al. 2011). Nevertheless, the use of everyday-type scenarios is undoubtedly of greater ecological validity than more traditional abstract tasks, and the clinical evidence suggests that reduced pro-social behaviour is likely to characterise everyday performance in ASD.

Tasks such as ‘Above and Beyond’ can help us to appreciate the nuances of social deficits, which in turn can be used to guide the focus of future interventions from an applied point of view. Individuals with high-functioning ASD may rely on compensatory strategies (Frith 2004; Hill and Frith 2003) such as the application of learned social rules to alleviate perspective-taking deficits. Whilst the use of such strategies may in some circumstances effectively mask aspects of social impairment (Frith 2003, 2004), the inflexible application of learned social rules may also result in socially inappropriate responses in those with ASD (Channon et al. 2010; Howlin 2004). In the context of impaired perspective-taking, social understanding in children with ASD may be acquired through deliberate effort, as compared to the intuitive and relatively effortless acquisition of typically developing children (Travis et al. 2001). Using tasks such as ‘Above and Beyond’ may inform training programmes which aim to improve social and communicative difficulties in those with ASD. Whilst a number of such programmes have been developed, these have concentrated on children and/or younger adolescents, and are thus often unsuited to high-functioning adults. In the past, training typically focused on ameliorating behavioural deficits, such as difficulties in turn-taking, conversational skills or limited eye contact (see e.g. Barry et al. 2003; Kamps et al. 1992; Marriage et al. 1995). The focus has since shifted to targeting component skills thought to underpin behavioural deficits, such as teaching perspective-taking (see e.g. Gray 1995; McGregor et al. 1998). Despite considerable attention to social skills training in people with ASD, relatively little is known about the efficacy of such programmes or the key ingredients for success (Mueser and Bellack 2007; Rao et al. 2008; Schreiber 2011; Reichow et al. 2013). It is difficult to achieve generalisation beyond the specific materials and environment used during the specific training programme (Howlin and Yates 1999). Thus, even if targeted skills improve, they do not easily translate to other settings, including the real-world environment. Materials such as the ‘Above and Beyond’ task could potentially be used to bridge the gap between training cognitive skills (perspective-taking and compensatory strategies) and navigating real-life situations. By combining understanding of what others may be thinking or feeling with behavioural choices for the participant, clear links could be made between social processing and principles of successful social interaction.

## Conclusion

In summary, this study has broadened understanding of social behaviour in ASD by examining pro-social behaviour in those with high versus low autistic traits. The high AQ group was found to behave less pro-socially overall than the low AQ group, and this pattern persisted when task demands were reduced. When faced with opportunities to behave pro-socially they produced spontaneous responses that were classified as less pro-social, and selected less pro-social courses of action when asked to choose from three options. The high AQ group also judged personal satisfaction for performing pro-social actions to be lower, although the groups did not differ in ratings of the characters’ satisfaction. Further studies based on tasks such as this could be instrumental both in informing our understanding and management of the everyday social deficits of those with ASD, and in shedding light on the cognitive and neural underpinnings of everyday social performance.

## Footnotes

1 The authors will provide copy of the ‘Above and Beyond’ task upon request.
